# Supplementary material for: Longevity is impacted by growth hormone action during early postnatal period
Source: eLife. 2017 Jul 4;6:e24059. doi: 10.7554/eLife.24059 (PMC5515575; doi:10.7554/eLife.24059)
Supplement: Supplementary file 2. — DOI: http://dx.doi.org/10.7554/eLife.24059.012 [file elife-24059-supp2.pdf]

Linear model for group=Dwarf, week=week1

The GLM Procedure

Class Level Information		
**Class**	**Levels**	**Values**
**sex**	2	Female Male
**treatment**	2	GH Saline

**Number of Observations Read**	54
**Number of Observations Used**	54

Linear model for group=Dwarf, week=week1

The GLM Procedure

Dependent Variable: day_alive day alive

Source	DF	Sum of Squares	Mean Square	F Value	Pr > F
**Model**	2	194158.150	97079.075	1.95	0.1534
**Error**	51	2545321.775	49908.270		
**Corrected Total**	53	2739479.926			

R-Square	Coeff Var	Root MSE	day_alive Mean
0.070874	24.23108	223.4016	921.9630

Source	DF	Type I SS	Mean Square	F Value	Pr > F
**sex**	1	29889.9650	29889.9650	0.60	0.4426
**treatment**	1	164268.1854	164268.1854	3.29	0.0755

Source	DF	Type III SS	Mean Square	F Value	Pr > F
**sex**	1	27703.8190	27703.8190	0.56	0.4597
**treatment**	1	164268.1854	164268.1854	3.29	0.0755

Linear model for group=Dwarf, week=week1

The GLM Procedure
Least Squares Means

treatment	day_alive LSMEAN
GH	877.774591
Saline	988.401076

Linear model for group=Normal, week=week1

The GLM Procedure

Class Level Information		
**Class**	**Levels**	**Values**
**group**	1	Normal
**sex**	2	Female Male
**treatment**	2	GH Saline

**Number of Observations Read**	80
**Number of Observations Used**	80

Linear model for group=Normal, week=week1

The GLM Procedure

Dependent Variable: day_alive   day alive

Source	DF	Sum of Squares	Mean Square	F Value	Pr > F
Model	2	10976.466	5488.233	0.17	0.8457
Error	77	2515421.522	32667.812		
Corrected Total	79	2526397.988			

R-Square	Coeff Var	Root MSE	day_alive Mean
0.004345	25.86144	180.7424	698.8875

Source	DF	Type I SS	Mean Square	F Value	Pr > F
sex	1	10829.05568	10829.05568	0.33	0.5665
treatment	1	147.40993	147.40993	0.00	0.9466

Source	DF	Type III SS	Mean Square	F Value	Pr > F
sex	1	10932.59318	10932.59318	0.33	0.5646
treatment	1	147.40993	147.40993	0.00	0.9466

Linear model for group=Normal, week=week1

The GLM Procedure
Least Squares Means

treatment	day_alive LSMEAN
**GH**	701.496069
**Saline**	698.736053

Linear model for group=Dwarf, sex=Male, week=week1

The GLM Procedure

Class Level Information		
**Class**	**Levels**	**Values**
**group**	1	Dwarf
**sex**	1	Male
**treatment**	2	GH Saline

**Number of Observations Read**	35
**Number of Observations Used**	35

Linear model for group=Dwarf, sex=Male, week=week1

The GLM Procedure

Dependent Variable: day_alive day alive

Source	DF	Sum of Squares	Mean Square	F Value	Pr > F
Model	1	251824.076	251824.076	6.54	0.0153
Error	33	1270370.095	38496.063		
Corrected Total	34	1522194.171			

R-Square	Coeff Var	Root MSE	day_alive Mean
0.165435	21.68892	196.2041	904.6286

Source	DF	Type I SS	Mean Square	F Value	Pr > F
treatment	1	251824.0760	251824.0760	6.54	0.0153

Source	DF	Type III SS	Mean Square	F Value	Pr > F
treatment	1	251824.0760	251824.0760	6.54	0.0153

Linear model for group=Dwarf, sex=Male, week=week1

The GLM Procedure
Least Squares Means

treatment	day_alive LSMEAN
GH	826.789474
Saline	997.062500

Linear model for group=Dwarf, sex=Female, week=week1

The GLM Procedure

Class Level Information		
**Class**	**Levels**	**Values**
**sex**	1	Female
**treatment**	2	GH Saline

**Number of Observations Read**	19
**Number of Observations Used**	19

Linear model for group=Dwarf, sex=Female, week=week1

The GLM Procedure

Dependent Variable: day_alive   day alive

Source	DF	Sum of Squares	Mean Square	F Value	Pr > F
Model	1	7.467	7.467	0.00	0.9919
Error	17	1187388.322	69846.372		
Corrected Total	18	1187395.789			

R-Square	Coeff Var	Root MSE	day_alive Mean
0.000006	27.70585	264.2846	953.8947

Source	DF	Type I SS	Mean Square	F Value	Pr > F
treatment	1	7.46725146	7.46725146	0.00	0.9919

Source	DF	Type III SS	Mean Square	F Value	Pr > F
treatment	1	7.46725146	7.46725146	0.00	0.9919

Linear model for group=Dwarf, sex=Female, week=week1

The GLM Procedure
Least Squares Means

treatment	day_alive LSMEAN
**GH**	953.300000
**Saline**	954.555556

Linear model for group=Normal, sex=Male, week=week1

The GLM Procedure

Class Level Information		
**Class**	**Levels**	**Values**
**group**	1	Normal
**sex**	1	Male
**treatment**	2	GH Saline

**Number of Observations Read**	36
**Number of Observations Used**	36

Linear model for group=Normal, sex=Male, week=week1

The GLM Procedure

Dependent Variable: day_alive   day alive

Source	DF	Sum of Squares	Mean Square	F Value	Pr > F
Model	1	7334.445	7334.445	0.15	0.6989
Error	34	1638986.305	48205.480		
Corrected Total	35	1646320.750			

R-Square	Coeff Var	Root MSE	day_alive Mean
0.004455	30.84755	219.5575	711.7500

Source	DF	Type I SS	Mean Square	F Value	Pr > F
treatment	1	7334.444805	7334.444805	0.15	0.6989

Source	DF	Type III SS	Mean Square	F Value	Pr > F
treatment	1	7334.444805	7334.444805	0.15	0.6989

Linear model for group=Normal, sex=Male, week=week1

The GLM Procedure
Least Squares Means

treatment	day_alive LSMEAN
GH	729.642857
Saline	700.363636

Linear model for group=Normal, sex=Female, week=week1

The GLM Procedure

Class Level Information		
**Class**	**Levels**	**Values**
**group**	1	Normal
**sex**	1	Female
**treatment**	2	GH Saline

**Number of Observations Read**	44
**Number of Observations Used**	44

Linear model for group=Normal, sex=Female, week=week1

The GLM Procedure

Dependent Variable: day_alive   day alive

Source	DF	Sum of Squares	Mean Square	F Value	Pr > F
**Model**	1	3598.2576	3598.2576	0.17	0.6782
**Error**	42	865649.9242	20610.7125		
**Corrected Total**	43	869248.1818			

R-Square	Coeff Var	Root MSE	day_alive Mean
0.004140	20.85588	143.5643	688.3636

Source	DF	Type I SS	Mean Square	F Value	Pr > F
**treatment**	1	3598.257608	3598.257608	0.17	0.6782

Source	DF	Type III SS	Mean Square	F Value	Pr > F
**treatment**	1	3598.257608	3598.257608	0.17	0.6782

Linear model for group=Normal, sex=Female, week=week1

The GLM Procedure
Least Squares Means

treatment	day_alive LSMEAN
**GH**	680.480000
**Saline**	698.736842

Linear model for group=Dwarf, week=week2

The GLM Procedure

Class Level Information		
**Class**	**Levels**	**Values**
**group**	1	Dwarf
**sex**	2	Female Male
**treatment**	2	GH Saline

**Number of Observations Read**	61
**Number of Observations Used**	61

Linear model for group=Dwarf, week=week2

The GLM Procedure

Dependent Variable: day_alive day alive

Source	DF	Sum of Squares	Mean Square	F Value	Pr > F
**Model**	2	354094.592	177047.296	6.22	0.0036
**Error**	58	1651377.638	28472.028		
**Corrected Total**	60	2005472.230			

R-Square	Coeff Var	Root MSE	day_alive Mean
0.176564	17.98489	168.7366	938.2131

Source	DF	Type I SS	Mean Square	F Value	Pr > F
**sex**	1	109779.2840	109779.2840	3.86	0.0544
**treatment**	1	244315.3076	244315.3076	8.58	0.0048

Source	DF	Type III SS	Mean Square	F Value	Pr > F
**sex**	1	158230.7877	158230.7877	5.56	0.0218
**treatment**	1	244315.3076	244315.3076	8.58	0.0048

Linear model for group=Dwarf, week=week2

The GLM Procedure
Least Squares Means

treatment	day_alive LSMEAN
GH	880.49322
Saline	1008.50104

Linear model for group=Normal, week=week2

The GLM Procedure

Class Level Information		
**Class**	**Levels**	**Values**
**group**	1	Normal
**sex**	2	Female Male
**treatment**	2	GH Saline

**Number of Observations Read**	62
**Number of Observations Used**	62

Linear model for group=Normal, week=week2

The GLM Procedure

Dependent Variable: day_alive   day alive

Source	DF	Sum of Squares	Mean Square	F Value	Pr > F
**Model**	2	379672.278	189836.139	6.88	0.0021
**Error**	59	1628572.319	27602.921		
**Corrected Total**	61	2008244.597			

R-Square	Coeff Var	Root MSE	day_alive Mean
0.189057	23.63047	166.1413	703.0806

Source	DF	Type I SS	Mean Square	F Value	Pr > F
**sex**	1	6962.0806	6962.0806	0.25	0.6174
**treatment**	1	372710.1970	372710.1970	13.50	0.0005

Source	DF	Type III SS	Mean Square	F Value	Pr > F
**sex**	1	8.4309	8.4309	0.00	0.9861
**treatment**	1	372710.1970	372710.1970	13.50	0.0005

Linear model for group=Normal, week=week2

The GLM Procedure
Least Squares Means

treatment	day_alive LSMEAN
GH	611.057223
Saline	769.542006

Linear model for group=Dwarf, sex=Male, week=week2

The GLM Procedure

Class Level Information		
**Class**	**Levels**	**Values**
**group**	1	Dwarf
**sex**	1	Male
**treatment**	2	GH Saline

**Number of Observations Read**	36
**Number of Observations Used**	36

Linear model for group=Dwarf, sex=Male, week=week2

The GLM Procedure

Dependent Variable: day_alive   day alive

Source	DF	Sum of Squares	Mean Square	F Value	Pr > F
Model	1	153230.9722	153230.9722	6.65	0.0144
Error	34	783727.3333	23050.8039		
Corrected Total	35	936958.3056			

R-Square	Coeff Var	Root MSE	day_alive Mean
0.163541	16.81598	151.8249	902.8611

Source	DF	Type I SS	Mean Square	F Value	Pr > F
treatment	1	153230.9722	153230.9722	6.65	0.0144

Source	DF	Type III SS	Mean Square	F Value	Pr > F
treatment	1	153230.9722	153230.9722	6.65	0.0144

Linear model for group=Dwarf, sex=Male, week=week2

The GLM Procedure
Least Squares Means

treatment	day_alive LSMEAN
GH	825.666667
Saline	958.000000

Linear model for group=Dwarf, sex=Female, week=week2

The GLM Procedure

Class Level Information		
**Class**	**Levels**	**Values**
**group**	1	Dwarf
**sex**	1	Female
**treatment**	2	GH Saline

**Number of Observations Read**	25
**Number of Observations Used**	25

Linear model for group=Dwarf, sex=Female, week=week2

The GLM Procedure

Dependent Variable: day_alive   day alive

Source	DF	Sum of Squares	Mean Square	F Value	Pr > F
Model	1	91480.5945	91480.5945	2.43	0.1330
Error	23	867254.0455	37706.6976		
Corrected Total	24	958734.6400			

R-Square	Coeff Var	Root MSE	day_alive Mean
0.095418	19.63181	194.1821	989.1200

Source	DF	Type I SS	Mean Square	F Value	Pr > F
treatment	1	91480.59455	91480.59455	2.43	0.1330

Source	DF	Type III SS	Mean Square	F Value	Pr > F
treatment	1	91480.59455	91480.59455	2.43	0.1330

Linear model for group=Dwarf, sex=Female, week=week2

The GLM Procedure
Least Squares Means

treatment	day_alive LSMEAN
GH	935.50000
Saline	1057.36364

Linear model for group=Normal, sex=Male, week=week2

The GLM Procedure

Class Level Information		
**Class**	**Levels**	**Values**
**group**	1	Normal
**sex**	1	Male
**treatment**	2	GH Saline

**Number of Observations Read**	31
**Number of Observations Used**	31

Linear model for group=Normal, sex=Male, week=week2

The GLM Procedure

Dependent Variable: day_alive   day alive

Source	DF	Sum of Squares	Mean Square	F Value	Pr > F
**Model**	1	624639.642	624639.642	18.72	0.0002
**Error**	29	967613.132	33365.970		
**Corrected Total**	30	1592252.774			

R-Square	Coeff Var	Root MSE	day_alive Mean
0.392299	25.59469	182.6635	713.6774

Source	DF	Type I SS	Mean Square	F Value	Pr > F
**treatment**	1	624639.6424	624639.6424	18.72	0.0002

Source	DF	Type III SS	Mean Square	F Value	Pr > F
**treatment**	1	624639.6424	624639.6424	18.72	0.0002

Linear model for group=Normal, sex=Male, week=week2

The GLM Procedure
Least Squares Means

treatment	day_alive LSMEAN
GH	522.272727
Saline	818.950000

Linear model for group=Normal, sex=Female, week=week2

The GLM Procedure

Class Level Information		
**Class**	**Levels**	**Values**
**group**	1	Normal
**sex**	1	Female
**treatment**	2	GH Saline

**Number of Observations Read**	31
**Number of Observations Used**	31

Linear model for group=Normal, sex=Female, week=week2

The GLM Procedure

Dependent Variable: day_alive   day alive

Source	DF	Sum of Squares	Mean Square	F Value	Pr > F
**Model**	1	7833.0586	7833.0586	0.57	0.4578
**Error**	29	401196.6833	13834.3684		
**Corrected Total**	30	409029.7419			

R-Square	Coeff Var	Root MSE	day_alive Mean
0.019150	16.98517	117.6196	692.4839

Source	DF	Type I SS	Mean Square	F Value	Pr > F
**treatment**	1	7833.058602	7833.058602	0.57	0.4578

Source	DF	Type III SS	Mean Square	F Value	Pr > F
**treatment**	1	7833.058602	7833.058602	0.57	0.4578

Linear model for group=Normal, sex=Female, week=week2

The GLM Procedure
Least Squares Means

treatment	day_alive LSMEAN
**GH**	676.066667
**Saline**	707.875000
